# Impact of mild-moderate mitral regurgitation on outcomes of isolated aortic valve replacement

**DOI:** 10.1186/1749-8090-10-S1-A180

**Published:** 2015-12-16

**Authors:** Rong Hui (Misté) Chia, Julian A Smith, Rory SJ Wolfe, Aubrey A Almeida

**Affiliations:** 1 Department of Cardiothoracic Surgery, Monash Health, Melbourne, Victoria, 3168, Australia; 2 Department of Epidemiology and Preventive Medicine, Monash University, Melbourne, Victoria, 3004, Australia

## Background/Introduction

The impact of mitral regurgitation (MR) severity on patients undergoing aortic valve replacement (AVR) for aortic stenosis remains unclear.

## Aims/Objectives

This study evaluated the effects of mild or moderate MR on outcomes of isolated AVR for aortic stenosis.

## Method

Clinical outcomes evaluated were postoperative complications; length of stay in intensive care unit (ICU); 30-day and late mortality; and the degree of MR improvement on echocardiograms after AVR. MR severity was defined according to the European Association of Echocardiography recommendations. Medium-term functional outcome was assessed using the Short Form-36 quality of life (QoL) questionnaire.

## Results

Eighty-nine patients received isolated AVR for significant aortic stenosis from August 2008 to September 2014, of which 53 patients had co-existing mild MR while 36 had moderate MR. Both groups were similar prior to surgery, except in the incidence of concomitant aortic regurgitation (28% versus 17%). Median follow-up time for postoperative echocardiograms and QoL assessment were 1 and 3 years respectively.

The odds of postoperative complications were greater in the moderate MR patients although this may have been a chance observation (OR, 2.3; p = 0.3). Mild MR patients had fewer postoperative complications (mean of 2 ± 2 versus 3 ± 3 complications; p = 0.04). There was no significant difference in the odds of mean duration of ICU-stay (mean 3 ± 3 versus 4 ± 4 days; p = 0.4) or 30-day mortality between groups (OR, 1.6; p = 0.5). Difference in late-mortality was insignificant (HR, 1.2; P = 0.5). Based on available postoperative echocardiograms, mild MR (n = 37) worsened in MR grade by 0.1 ± 0.5 whereas moderate MR (n = 27) improved by 0.4 ± 0.6 in MR grade after AVR (p = 0.01). Fewer patients with mild MR made an improvement in MR grade postoperatively (27% versus 59%). Only 79% and 72% of the patients in mild and moderate MR groups respectively were alive at the time of survey. At follow-up, both groups had similar QoL.

## Discussion/Conclusion

Clinical and medium-term functional outcomes were similar in patients with mild or moderate MR undergoing isolated AVR.

